# Tralokinumab pharmacokinetics and tolerability when administered by different subcutaneous injection methods and rates

**DOI:** 10.5414/CP203023

**Published:** 2017-06-07

**Authors:** Meena Jain, Diane Doughty, Corbin Clawson, Xiaobai Li, Nicholas White, Balaji Agoram, René van der Merwe

**Affiliations:** 1 Clinical Development, MedImmune, Cambridge, UK,; 2 Drug Delivery and Device Development,; 3 Clinical Biostatistics, MedImmune, Gaithersburg, MD, USA,; 4 Clinical Pharmacology, Drug Metabolism and Pharmacokinetics, MedImmune, Cambridge, UK, and; 5 Clinical Pharmacology, Drug Metabolism and Pharmacokinetics, MedImmune, Mountain View, CA, USA

**Keywords:** asthma, pharmacokinetics, subcutaneous, tolerability, tralokinumab

## Abstract

Objective: Tralokinumab, administered as two 1-mL subcutaneous injections every 2 weeks, at the target dose 300 mg, has been shown to improve lung function in patients with asthma. This study evaluated the pharmacokinetic (PK) and tolerability profile of tralokinumab 300 mg when administered by different rates of subcutaneous injection, as part of a pilot investigation of new injection regimens. Methods: This phase I study randomized 60 healthy adults to receive 300 mg tralokinumab, as two 1-mL subcutaneous injections, each delivered over 10 seconds, or one 2-mL injection delivered over 10 seconds (12 mL/min), 1 minute (2 mL/min), or 12 minutes (0.167 mL/min). Results: No differences in the PK profile of tralokinumab were observed between cohorts. Immediately following injection, injection-site pain intensity (mean (SD)) was lowest following 0.167 mL/min injection (5.1 mm (8.0) via visual analog scale (VAS)) and greatest following 12 mL/min injection (41 mm (27.7) via VAS); with mean injection-site pruritus intensity low for all participants. Two types of local injection-site reactions were observed: erythema (58.3%) and hematoma/bleeding (18.3%). All treatment-emergent adverse events were mild. Conclusions: Tralokinumab 300 mg is well tolerated, with comparable PK, when administered by a single 2-mL injection at different rates of subcutaneous injection vs. two 1-mL injections.

**Supplemental material is available for free at: www.clinpharmacol.com Vol. 55, issue 7.**

## Introduction 

Tralokinumab is a human monoclonal antibody (mAb) of the immunoglobulin G_4_ (IgG_4_) subclass that potently and specifically neutralizes interleukin 13 (IL-13) by preventing its interaction with the receptors, IL-13Rα1 and IL-13Rα2 [1]. IL-13 is one of the key mediators in the pathogenesis of asthma [2]; thus, inhibition of IL-13 by tralokinumab offers a rational approach to the treatment of this disease [1]. Results from phase II studies in adult patients with uncontrolled severe asthma suggest that the addition of tralokinumab at a dose of 300 mg every 2 weeks (Q2W) alongside the patient’s current asthma therapy may provide clinical improvements in this patient population [3, 4]. These clinical improvements were demonstrated by an improvement in lung function, as well as an acceptable safety and tolerability profile [3, 4]. 

Tralokinumab is currently being investigated for the treatment of asthma in multiple phase III studies [5, 6, 7]. In these studies, tralokinumab is administered as two 1-mL subcutaneous (SC) injections of 150 mg/mL either Q2W or every 4 weeks (Q4W). However, the ability to administer tralokinumab at the target therapeutic dose of 300 mg via a single 2-mL SC injection may be preferable to some patients. 

It is important to determine whether the pharmacokinetic (PK) profile of the required dose of 300 mg tralokinumab is affected when delivered via a different dosing regimen (i.e., a single injection vs. multiple injections, or a fast vs. slow flow rate), as this may have therapeutic consequences with respect to safety and efficacy. For the delivery of 300 mg tralokinumab in a single 2-mL injection to be feasible, the PK temporal profile should be similar to that of two 1-mL injections, and delivery must be tolerable for the patient. Studies involving the administration of a single large volume (up to 3.5 mL) SC injection of placebo solutions, at the anticipated viscosities of biotherapeutic agents, have demonstrated favorable safety profiles in terms of pain, tolerability, and treatment-emergent adverse events (TEAEs) [8, 9, 10]. However, the effect on PK of larger volumes of an active substance delivered at different flow rates has not been reported. Demonstration that the PK of 300 mg tralokinumab is not affected by administration as a 2-mL injection or by a reasonable range of flow rates could provide the basis for the development of new delivery devices for this molecule. 

Currently, most SC injection volumes of therapeutic compounds, including mAbs, are limited to 1 mL, as volumes greater than 1 mL have been associated with tolerability issues such as increased injection pain, high SC back pressure from the tissue site, site leakage, and injection-site reactions [11, 12]. Therefore, in some instances, in order to deliver the therapeutic doses required, multiple injections may be necessary. Self-administration of SC injections of up to 1 mL is clinically acceptable for therapeutic mAb products, with several products commercially available [13, 14, 15, 16, 17]. Where greater doses are required to achieve a therapeutic effect, mAbs may also be formulated at concentrations of > 100 mg/mL. However, the resulting increase in viscosity may affect delivery by SC injection [18, 19], particularly when the delivery device requires a fast injection rate [20]. Conversely, one recent study has presented data that suggests relatively large SC (up to 3 mL) injections are well tolerated, regardless of injection flow rate (within 10 seconds) or fluid viscosity (up to 15 – 20 cP), when injected into the abdomen [9]. Other studies of high-volume, high-viscosity SC injections (such as immunoglobulin replacement therapy) have also demonstrated that SC tissue can accommodate volumes larger than 1 mL with good tolerability when the flow rate and delivery method are optimized [8, 9, 10, 21, 22, 23]. Clearly, tolerability factors – alongside the PK profile – need to be considered as part of the development of injection strategies and devices that may administer tralokinumab by different methods (i.e., a single injection vs. multiple injections) and/or rate of SC injection. 

As part of a pilot investigation, we describe the results of an open-label, parallel-group study to explore the PK and tolerability of tralokinumab 300 mg when administered by different rates of SC injection and as a single 2-mL injection vs. two 1-mL injections. 

## Methods 

### Study objective

The primary objective was to evaluate the PK profile of a single SC dose of 300 mg tralokinumab, delivered as a 2-mL injection at different flow rates to healthy adult volunteers. Secondary objectives were to determine the local tolerability (as assessed by injection-site pain and pruritus intensity and injection-site reactions), overall safety, and immunogenicity profile of delivering 300 mg tralokinumab as either a single SC dose or as a 2-mL injection at different flow rates. 

### Study design and participants

**Figure 1.**
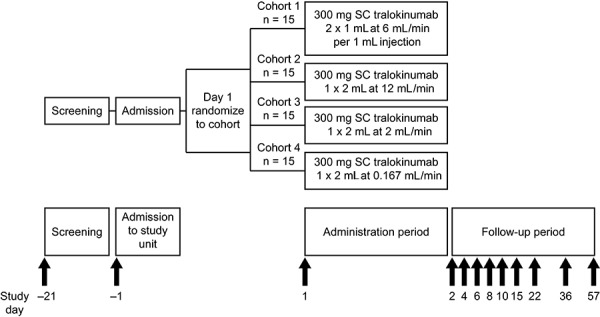
Study design. SC = subcutaneous.

In this phase I, open-label, single-blind, parallel-group study (NCT02085473), healthy males and females were enrolled from one site in the USA. Participants aged 19 – 65 years with a body mass index of 19 – 30 kg/m^2^ were included in the study. For key inclusion and exclusion criteria see Supplemental Table 1. Enrolled participants were randomized in a 1 : 1 : 1 : 1 ratio to one of four cohorts. See Figure 1 for study design. 

A screening visit was performed in the 21 days prior to dosing. Participants were admitted to the study unit on day –1. Due to the potential for sex differences in pain perception and sensitivity [24, 25], randomization was stratified by sex, with a minimum of 5 males and 5 females randomized to each cohort; additional participants were of either sex. Following administration of 300 mg tralokinumab on day 1, participants were discharged on the same day after all study procedures were completed, then followed up as outpatients for 57 days for safety, tolerability, immunogenicity, and PK sampling. 

**Figure 2.**
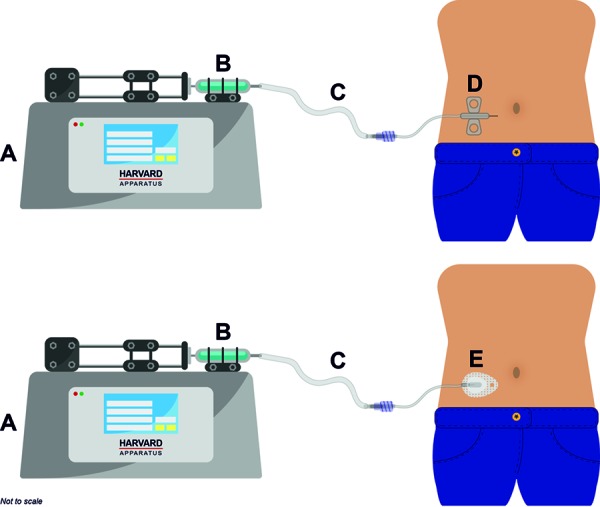
Experimental setup. The fluid path consisted of sterile, 510(k)-cleared components attached together via Luer connections. A Harvard syringe pump (HA3000W PHD Ultra infuse/withdraw syringe pump) (A), with a 10 mL plastic Luer-Lok™ syringe (BD) (B) and Microbore extension set (B. Braun) (C) attached were used to deliver tralokinumab at the required volume and flow rate. For cohorts 1 and 2, a 25-G × ½” Surflo^®^ winged infusion set with 8” tubing (Terumo) (rigid needle) mimicking a standard subcutaneous injection was used (D). For cohorts 3 and 4, the Animas Inset™ infusion system with a 6-mm, 25-G soft cannula was used to mimic a on body SC delivery system (E).

All injections were administered SC in the abdomen using a Harvard syringe pump (HA3000W PHD Ultra infuse/withdraw syringe pump; Instech Laboratories Inc., Plymouth Meeting, PA, USA), to ensure accuracy of flow rates and corresponding delivery times. The fluid path consisted of sterile, 510(k)-cleared components attached together via Luer connections. For cohort 1, two separate injection sites on the same side of the abdomen, spaced at least 3 cm apart, were used for each participant. For cohorts 2, 3, and 4, a single injection site was identified on the abdomen for each participant. For all cohorts, tralokinumab was delivered via a 10-mL plastic Luer-Lok™ syringe (BD; Franklin Lakes, NJ, USA), B. Braun microbore extension set, and an insertion set apparatus. For cohorts 1 and 2, a rigid needle, winged infusion set was selected (Terumo 25 G × ½” Surflo^®^ (Surflo^®^, Terumo, Somerset, USA)) with 8” tubing (Terumo, Liverpool, UK), to represent the insertion apparatus for a standard SC injection. For cohorts 3 and 4, a soft cannula insertion set was used (Animas Inset™ infusion system (Animas, Livingstone, UK) with a 6-mm, 25-G soft cannula) to mimic a typical on-body device (e.g., insulin pump therapy). See Figure 2 for a schematic representation of the experimental apparatus. 

Tralokinumab was delivered at the following times: cohort 1, 10 seconds for each 1-mL injection (i.e., 6 mL/min per injection); cohort 2, 10 seconds for a single 2-mL injection (i.e., 12 mL/min, or 1 mL/5 s); cohort 3, 1 minute for a single 2-mL injection (i.e., 2 mL/min, or 1 mL/30 s); cohort 4, 12 minutes for a single 2-mL injection (i.e., 0.167 mL/min, 1 mL/360 s). In cohorts 1 and 2, the delivery methods and delivery times were designed to mimic those typically used in routine SC administration by pre-filled syringes or autoinjector devices. Cohorts 3 and 4 were intended to simulate delivery rates of an on-body SC delivery system. For a summary of the different flow rates, see Table 1. 

### Study assessments

The primary endpoint for this study was to determine the PK profile of tralokinumab. Blood samples were taken for PK analyses immediately prior to tralokinumab administration and then on days 2, 4, 6, 8, 10, 15, 22, 36, and 57. Tralokinumab serum concentrations were quantified using a validated sandwich immunoassay as previously described [26]. 

Secondary endpoints were local injection-site pain intensity and injection-site pruritus, as measured by a 100-mm visual analog scale (VAS; where 0 mm = no pain/itch and 100 mm = worst imaginable pain/itch) immediately following tralokinumab injection and then at 10, 20, 30, and 60 minutes, and 2, 4, 8, 24, and 72 hours post injection. Local injection-site pain intensity was also recorded at 1 minute and 6 minutes during the administration of tralokinumab for cohort 4, due to the longer delivery time of the injection. For participants in cohort 1, an overall assessment of pain intensity and pruritus from both injection sites for each time point post injection was recorded. The presence of local injection-site reactions (including erythema, hematoma or bleeding, local warmth, swelling, and/or rash) was also recorded at the same time points as for injection-site pain intensity and pruritus, and included a measurement of the diameter of each injection-site reaction and an assessment of severity. Severity was graded from 1 to 5, representing mild, moderate, severe, life-threatening, and fatal. For detailed descriptions of how each adverse event (AE) or serious adverse event (SAE) was graded by severity, please see Supplemental Table 2. The assessment was performed by a blinded assessor immediately after administration for cohorts 2, 3, and 4. However, it was not possible for the assessor to be blinded to the treatment allocation of those participants in cohort 1, due to the presence of two injection sites. Furthermore, due to the variation in administration times, participants were not blinded in this study. 

Local injection-site reactions were recorded on a dedicated and standardized assessment questionnaire. Where there were differences in injection-site reactions between the two sites for participants in cohort 1, the most severe reaction was recorded. If it occurred, fluid leakage was measured immediately following administration by blotting the injection site with a pre-weighed absorbent material and the amount of leakage quantified by gravimetric analysis. Blood samples were collected to assess the presence of antidrug antibodies (ADA) against tralokinumab immediately prior to tralokinumab administration (day 1) and on day 57. 

Safety and tolerability of tralokinumab were assessed by monitoring the incidence of TEAEs and treatment-emergent serious adverse events (TESAEs), as well as clinical laboratory parameters, vital signs, and physical examinations throughout the study. 

As local injection-site reactions were recorded as a specific endpoint, they were only reported as AEs if they met one or more of the following criteria: They were an SAE, led to premature termination of the injection during tralokinumab administration, required concomitant medication or other medically important intervention, or had an impact on the general condition of the participant (as judged by the investigator). 

### Ethical approval

Informed consent was obtained from all study participants prior to the initiation of any study procedure. All study activities complied with Good Clinical Practice (GCP), guidelines of the International Conference on Harmonization (ICH), and all applicable regulatory requirements. 

All procedures performed in studies involving human participants were in accordance with the ethical standards of the institutional and/or national research committee and with the 1964 Helsinki declaration and its later amendments or comparable ethical standards. 

## Statistical analysis

*Sample size*

In a previous study (NCT01592396; data on file) the coefficient of variation (CV) was 37% for area under the serum concentration-time curve from zero to infinity (AUC_(0–∞)_) for tralokinumab. Assuming the same CV of 37% for AUC_(0–∞)_ across the cohorts, and using a two-sided, two-sample t-test at a significance level of 0.05, the sample size of 15 participants per cohort provided 84% power to detect a 1.5-fold difference in AUC_(0–∞)_ between cohort 1 and any of the other treatment cohorts. 

*Analysis populations and methods*

The as-treated population included all participants who received any amount of tralokinumab. Demographics and safety and tolerability endpoints were summarized based on the as-treated population. The PK population included all participants in the as-treated population with at least one detectable tralokinumab serum concentration and for whom the PK parameters could be adequately estimated. 

PK parameters included AUC_(0–∞)_, AUC to last observation (AUC_(0–t)_), apparent systemic clearance (CL/F), maximum observed concentration (C_max_), the time to C_max_ (t_max_), tralokinumab half-life (T_1/2_), and apparent terminal-phase volume of distribution (Vz/F). Parameter values were estimated for the PK population using non-compartmental models as implemented in Phoenix WinNonlin^®^ version 6.3 (Princeton, NJ, USA), a component of Phoenix version 1.3. Other safety/tolerability endpoints were analyzed for the as-treated population. For injection-site pain and pruritus, descriptive statistics were reported at each assessment time point by cohort. Wilcoxon rank-sum tests were used to compare VAS scores between cohort 1 and each of cohorts 2, 3, and 4. Local injection-site reactions were summarized using descriptive statistics by cohort. The safety profile of tralokinumab was assessed by summarizing AEs and TEAEs by cohort. AEs and TEAEs were also summarized by severity. Changes from baseline in laboratory measurements at each time point were summarized from the start of tralokinumab injection on day 1 until the end of the study (day 57). No multiplicity adjustment was made given the exploratory nature of the study. 

## Results 

### Participant disposition

**Figure 3.**
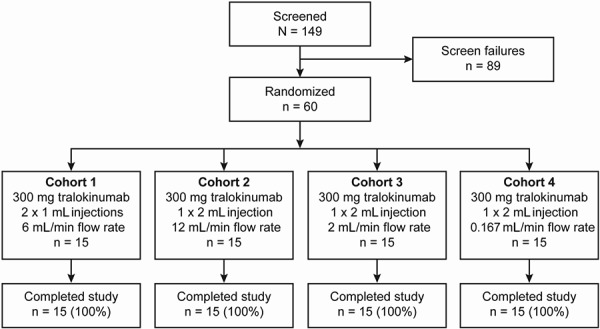
Participant disposition.

The study took place between March 19, 2014 (date first participant signed informed consent) and June 20, 2014 (date of last visit of last participant). A total of 149 healthy volunteers were screened for inclusion. Overall, 60 participants met the eligibility criteria and were randomized into the study, 15 participants per cohort. All participants received study medication and there were no discontinuations; all 60 participants completed the study (Figure 3). 

### Participant demographics

All 60 randomized participants were included in the as-treated population. Demographic characteristics were generally similar between the 4 cohorts (Table 2). Mean (standard deviation (SD)) age of the population was 38.3 (12.2) years with an overall equal number of male and female participants. 

### Pharmacokinetics evaluation

In total, 58 participants were included in the PK population. Three participants (1 from cohort 3 and 2 from cohort 4) had extensive leakage of tralokinumab, attributed to incorrect placement of the insertion set. Extensive leakage was defined as a measured leakage weight > 1 g, equivalent to ~ 50% of the injection volume (calculated as follows; tralokinumab solution weighs 2.092 g (based on a known density of 1.046 g/L), thus, 1 g represents 1 mL or ~ 50% of the injection volume). Of the 3 participants with extensive leakage, 2 (1 from cohort 3 and 1 from cohort 4) were excluded from the PK population because serum tralokinumab concentrations were below the level of detection at all PK sampling time points after dosing. The participant with extensive leakage who remained in the PK population was included because detectable serum concentrations of tralokinumab were observed. However, additional analyses excluding this participant were also performed on all PK parameters. 

**Figure 4.**
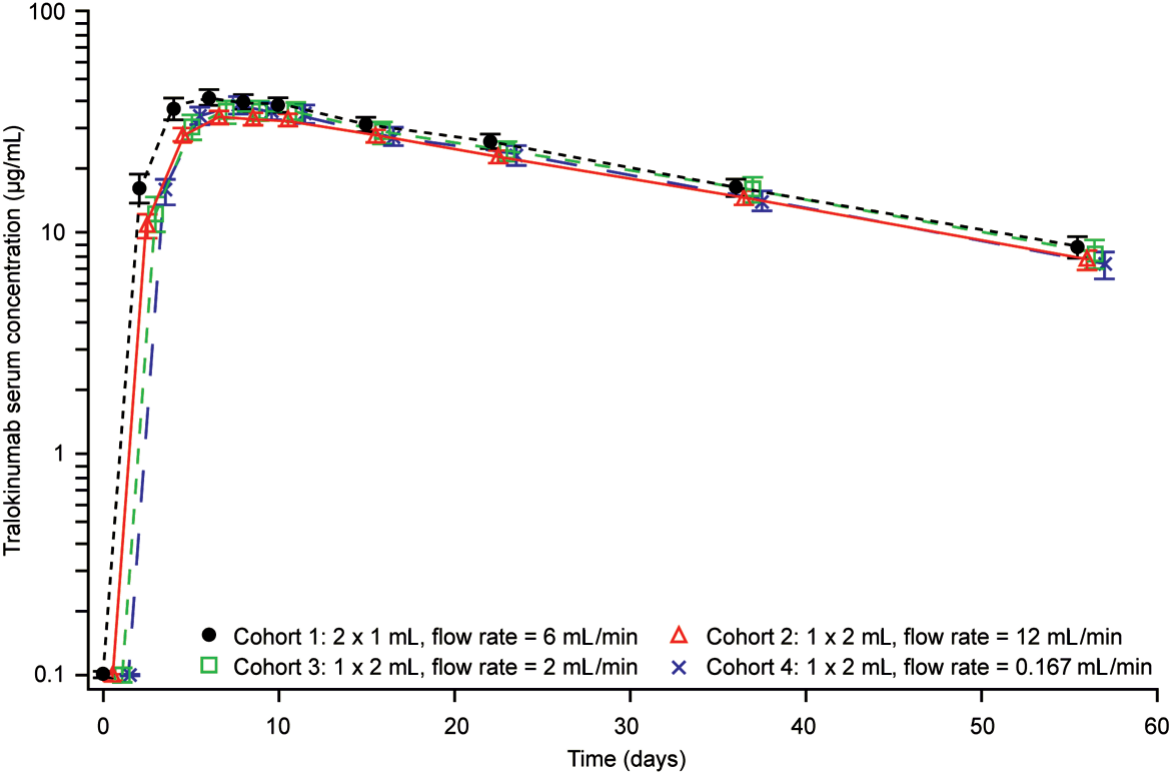
Mean (SD) serum concentration-time profiles of tralokinumab by cohort (PK population).^a ^PK = pharmacokinetic; SD = standard deviation. ^a^The participant in cohort 4 with extensive tralokinumab leakage was included in the patient population as detectable levels of tralokinumab were observed in the serum.

**Figure 5.**
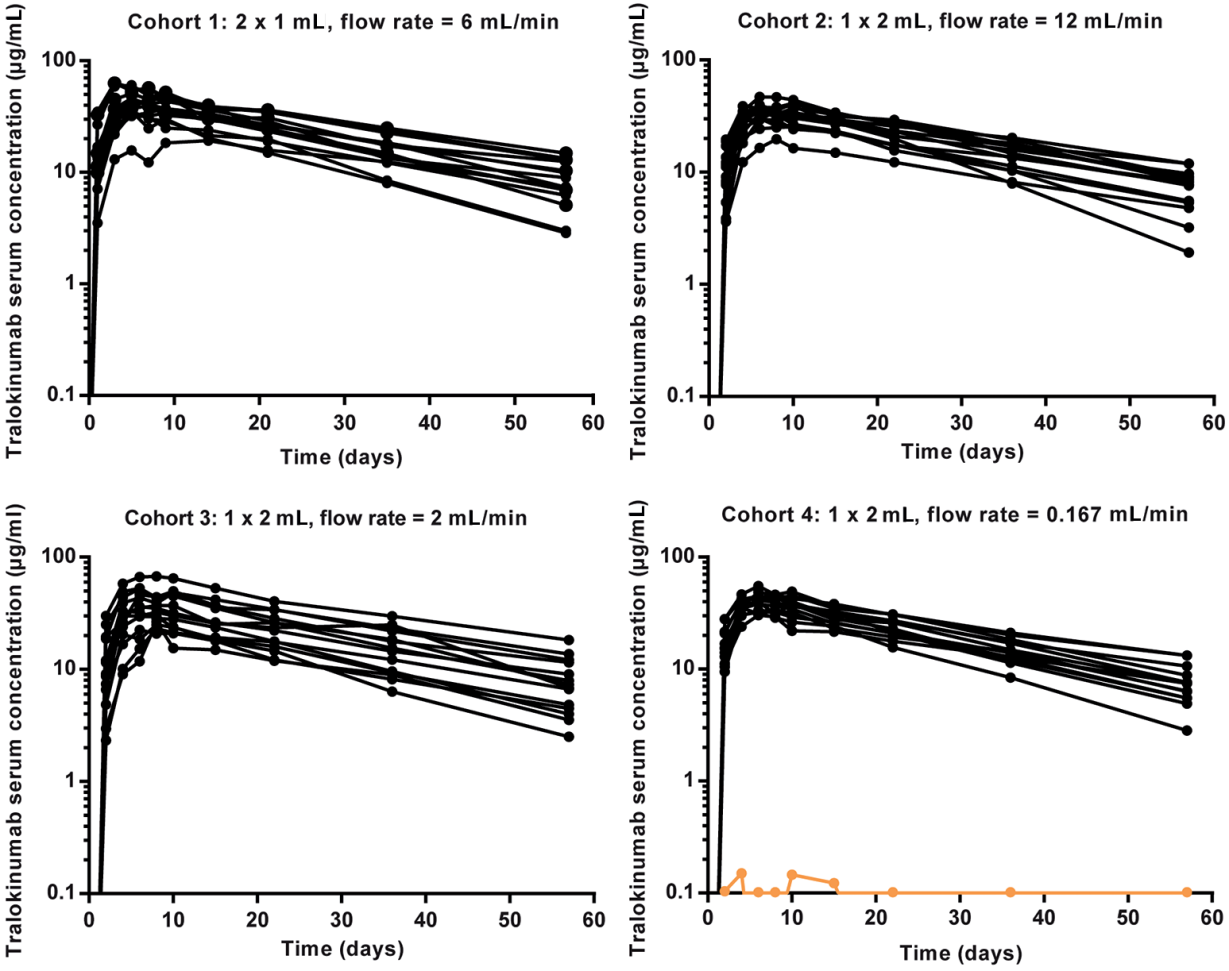
Individual serum concentration-time profiles of tralokinumab by cohort (PK population). In cohort 4, data for the participant with extensive leakage is represented with an orange line. PK = pharmacokinetic.

Mean tralokinumab serum concentration-time profiles were similar across the four cohorts (Figure 4). Individual PK profiles in each cohort are presented in Figure 5. A summary of the PK parameters can be found in Table 3. There were no statistically significant differences in any of the PK parameters between the cohorts. The area under the serum concentration curve (AUC_(0–∞)_) in cohorts 2 and 3 were similar (< 15% difference) compared with cohort 1 (Table 3). The ratio of AUC_(0–∞)_ for cohort 1 to that for cohort 4 (fold difference) was 1.6 for the PK population, but reduced to 1.1 when the participant with extensive leakage was excluded from cohort 4 (Table 3). No participants had confirmed positive ADAs to tralokinumab in the study. 

## Local tolerability

*Injection-site pain intensity*

Immediately after injection, the mean (SD) injection-site pain intensity was 21.9  mm (20.9) VAS for cohort 1, 41.0 mm (27.7) VAS for cohort 2 (p = 0.034), 17.7 mm (15.5) VAS for cohort 3 (p = 0.675), and 5.1 mm (8.0) VAS for cohort 4 (p = 0.002) (Supplemental Figure 1A). By 10 minutes post injection, mean injection-site pain intensity markedly decreased for all cohorts, with similar mean scores for all cohorts observed by 30 minutes and at subsequent assessments. Injection-site pain intensities > 50 mm on the VAS (approximating to a pain intensity rating of moderate-to-severe [27]), immediately following injection occurred in 2 participants (13.3%) in cohort 1, 6 participants (40.0%) in cohort 2, and no participants in cohorts 3 and 4. When moderate-to-severe pain intensity occurred, it decreased to < 50 mm on the VAS within 20 minutes for all participants. There were no notable differences in injection-site pain intensity between male and female participants in any of the cohorts at any time point following injection. 

*Injection-site pruritus intensity*

Overall, mean injection-site pruritus intensity was low for all cohorts immediately post injection (mean (SD) 4.8 mm (7.5) in cohort 1, 15.1 mm (20.2) in cohort 2 (p = 0.147), 4.0 mm (5.6) in cohort 3 (p = 0.841), and 6.9 mm (15.5) in cohort 4 (p = 0.507)) (Supplemental Figure 1B). Mean (SD) VAS injection-site pruritus intensity at 30 minutes post injection for participants in cohort 1 did reach statistical significance compared to cohort 4 (0.5 mm (0.7) VAS vs. 1.8 mm (1.8) VAS, respectively; p = 0.035); however, the level of intensity was low for both groups and the difference was not considered clinically meaningful. Injection-site pruritus intensities of > 50 mm VAS occurred in 2 participants in cohort 2 and 1 participant in cohort 4 immediately following injection, but these reduced to ≤ 5 mm VAS within 30 minutes post injection. 

*Local injection-site reactions*

A summary of injection-site reactions following injection of tralokinumab can be found in Table 4. The two types of local injection-site reactions observed over the 72-hour period post injection were erythema and hematoma or bleeding; all events were mild in severity. 

Overall, 7 participants experienced injection-site erythema from cohort 1, 5 from cohort 2, 12 from cohort 3, and 11 from cohort 4, within 72 hours of receiving the tralokinumab injection. Overall, injection-site erythema was resolved by 2 hours post injection for all cohorts. However, 1 mild injection-site erythema reaction of 5 mm in diameter was reported in a participant in cohort 3 at 24 hours following injection and was resolved by 72 hours post injection. The mean diameter of injection-site erythema was measured, being numerically highest for cohort 2 immediately post injection (mean (SD) 25.0 mm (15.0) for cohort 1, 37.5 mm (24.7) for cohort 2, 29.1 mm (11.1) for cohort 3, and 27.8 mm (18.6) for cohort 4). 

Injection-site hematoma or bleeding occurred sporadically following tralokinumab injection, with the highest incidence in cohort 4. Two participants experienced injection-site hematoma or bleeding from cohort 1, 1 from cohort 2, 3 from cohort 3, and 5 from cohort 4, within 72 hours of receiving the tralokinumab injection. The mean diameter of injection-site hematoma or bleeding was measured, and overall found to be numerically highest for cohort 4 (mean (SD) 2.8 mm (2.0) immediately post injection (no participant experienced injection-site hematoma or bleeding in cohorts 1, 2, and 3 at this time point)). One participant in both cohort 1 and cohort 2 reported a mild injection-site hematoma at 24 hours post injection; both events were resolved by 72 hours following injection. One mild injection-site hematoma or bleeding reaction of 20 mm in diameter was reported in 1 participant in cohort 3 at the 72-hours time point. This event was not followed up to resolution per protocol follow-up procedures, and was recorded as a protocol deviation. 

*Tralokinumab leakage*

Leakage of tralokinumab was observed at the injection site in 1 participant in cohort 3 and 3 participants in cohort 4. Of these 4 participants, 1 participant in cohort 3 and 2 participants in cohort 4 had extensive leakage (defined as a measured leakage weight > 1 g). Extensive tralokinumab leakage in these participants was attributed to human error, rather than to the volume of injection or flow rate, resulting from the incorrect placement of the insertion set (Animas Inset™ infusion system) used in cohorts 3 and 4. The incorrect placement resulted in the soft cannula assembly partially slipping off the needle and bending when inserted into the SC tissue. 

### Other safety and tolerability findings

There were no deaths, SAEs, or TEAEs that resulted in discontinuation of tralokinumab reported in this study. All reported TEAEs were mild in intensity. The proportions of participants having at least 1 TEAE were similar across the four cohorts. An overall summary of TEAEs is presented in Table 5. 

The most frequently reported TEAEs in the overall participant population were headache (16.7%), nasal congestion (5.0%), and rhinorrhea (5.0%). An injection-site reaction occurred in 1 participant (6.7%) from cohort 2 (injection-site pain). This was recorded as a TEAE due to its impact on the general condition of the participant, as judged by the investigator and was described as mild in severity. No clinically meaningful abnormalities in hematology, serum chemistry, or urinalysis laboratory values were observed. There were no clinically meaningful changes in vital signs from baseline to day 57. 

## Discussion 

This pilot study evaluated the PK profile, safety, and tolerability of a single SC dose of 300 mg tralokinumab, when delivered by a single 2-mL injection at different flow rates, compared with that observed with the currently used two 1-mL injections. Results showed no significant differences in the PK profile of tralokinumab, as summarized by relevant PK parameters, when compared across a range of injection flow rates given as a single 2-mL injection, and between methods (i.e., a single 2-mL injection vs. two 1 mL injections). Furthermore, the overall tolerability of a single 2-mL injection of 300 mg tralokinumab was generally consistent with that of two 1-mL injections, particularly when delivered at slow flow rates (0.167 – 2 mL/min). 

No statistically significant differences were seen in any of the measured PK parameters, which is consistent with the relatively slow absorption time of tralokinumab after SC administration. This was evidenced by the comparable t_max_ (time to C_max_ (maximum concentration)) value of 6 – 8 days in the four cohorts analyzed. To our knowledge, this is the first time PK data have been compared for a mAb therapy when delivered at varying flow rates and dosing regimens (i.e., a single injection vs. multiple injections, or a fast vs. slow flow rate). These findings illustrate that PK was not affected by the delivery methods and injection flow rates investigated, and help support further development of injection devices to deliver tralokinumab. The lack of difference was expected as, following SC administration, absorption of antibodies occurs through lymphatic flow over multiple days [28]. Therefore, changes of a few seconds to minutes in the SC administration rate are unlikely to impact the overall PK profile. 

In terms of tolerability, delivery of 300 mg tralokinumab as a 2-mL injection at slow flow rates was generally associated with lower injection-site pain intensity compared with that observed following two 1-mL injections. However, this study was not powered to detect significant differences in pain intensity, and the findings require confirmation in larger, blinded, controlled clinical studies. It must also be noted that there is considerable variability associated with pain assessments due to the subjective nature of this parameter [29, 30, 31]. Nevertheless, overall, the pain outcomes observed in this study are generally consistent with those seen in previous published work showing favorable tolerability profiles, in terms of pain- and injection-site reactions, following administration of a large volume (up to 3.5 mL) SC injection of a viscous solution (up to 20 cP) [8, 9, 10]. 

In addition to the PK and pain-intensity findings observed in the current study, the mean intensity of injection-site pruritus was low at all time points post injection and generally similar between all cohorts. Erythema and hematoma or bleeding were the only types of local injection-site reactions reported, and all were mild in severity. Both erythema and hematoma or bleeding were reported at a higher incidence in cohorts 3 and 4 compared with cohorts 1 and 2. However, these injection-site reactions may have been related to the soft cannula insertion set rather than the delivery method, although this requires further investigation. In particular, the adhesive required to apply the soft cannula to the skin could have caused an increase in reddening and itching around the injection site. The longer residence time of the cannula in the skin could have also been the cause of the increase in hematoma or bleeding seen at the injection site in cohort 3 and, particularly, cohort 4, rather than the volume or flow rate of the injection. 

Significant injection-site leakage was only observed in 4 participants and was attributed to incorrect application of the insertion set, rather than to the volume of injection or flow rate. This suggests the adequate accommodation of large volume solutions (up to 2 mL) by the SC tissues, even at the highest flow rate tested (12 mL/min). 

The overall safety profile of 300 mg tralokinumab SC was favorable, and was consistent with other studies of this molecule in a healthy population [26, 32]. All TEAEs were mild in severity, and no SAEs, discontinuations, or deaths were reported. 

A limitation of this study is related to the open-label exploratory design and the lack of a study control. As such, the assessors, who evaluated local reactions to administration of tralokinumab, could not be blinded to the treatment allocation of participants in cohort 1. The large variations in delivery flow rate and the difference in number of injections received also prevented blinding of the participants to the treatment received. As different delivery apparatus were used for cohorts 1 and 2 (rigid needle) and for cohorts 3 and 4 (soft cannula) to mimic representative delivery systems, caution is required when directly comparing the tolerability data from these groups. Finally, the small sample size prevented any meaningful statistical comparisons across cohorts with respect to the secondary endpoints, and no correction for multiple testing was applied due to the exploratory nature of the study. 

## Conclusion 

The results from this exploratory study demonstrated comparable PK and tolerability when tralokinumab is delivered at a dose of 300 mg via a single 2-mL SC injection at different flow rates, vs. two 1-mL injections. Our findings therefore support the feasibility of administration of the therapeutic dose of tralokinumab as a single 2-mL injection, potentially via a device such as an on-body delivery system or autoinjector, and warrant further investigation. 

## Acknowledgments 

This study was sponsored by MedImmune, a member of the AstraZeneca Group. We would like to acknowledge Roja Narwal for providing testing support (compatibility of line materials with tralokinumab) ahead of the study, William Lambert for providing support during the design of the study, Tao Yang for assistance with statistical programming, and Wessel de Graaf for study management. 

We would also like to acknowledge the study participants and study personnel at Celerion who participated in this study. We thank Rachel Cicchelli, PhD, from QXV Communications, an Ashfield Company, Macclesfield, UK, who provided medical writing support funded by AstraZeneca, Cambridge, UK, in accordance with Good Publication Practice (GPP3) guidelines (http://www.ismpp.org/gpp3). 

## Conflict of interest 

All authors are employees of MedImmune, the sponsor of this study. 

**Table 1.** Regimens for delivery of tralokinumab.

Cohort number	Number of injections and volume	Needle type	Flow rate	Delivery time
1	2 × 1 mL	Rigid	6 mL/min	10 s/injection
2	1 × 2 mL	Rigid	12 mL/min	10 s
3	1 × 2 mL	Soft cannula	2 mL/min	1 min
4	1 × 2 mL	Soft cannula	0.167 mL/min	12 min

**Table 2.** Participant demographics (as-treated population).

Population	Tralokinumab 300 mg				
	2 × 1 mL SC injections	1 × 2 mL SC injection			
	Cohort 1 6 mL/min n = 15	Cohort 2 12 mL/min n = 15	Cohort 3 2 mL/min n = 15	Cohort 4 0.167 mL/min n = 15	Total N = 60
Age (years)					
Mean (SD)	36.5 (9.5)	39.7 (13.5)	38.5 (14.1)	38.4 (12.3)	38.3 (12.2)
Sex, n (%)					
Male	8 (53.3)	8 (53.3)	7 (46.7)	7 (46.7)	30 (50.0)
Female	7 (46.7)	7 (46.7)	8 (53.3)	8 (53.3)	30 (50.0)
BMI (kg/m^2^)					
Mean (SD)	24.8 (2.5)	26.4 (2.4)	26.0 (3.3)	26.8 (2.5)	26.0 (2.7)

BMI = body mass index; SC = subcutaneous; SD = standard deviation.

**Table 3.** Tralokinumab pharmacokinetic parameters (PK population).

Parameter (geometric mean unless stated)	Tralokinumab 300 mg				
	2 × 1 mL SC injections	1 × 2 mL SC injection			
	Cohort 1 6 mL/min n = 15	Cohort 2 12 mL/min n = 15	Cohort 3 2 mL/min n = 14	Cohort 4 0.167 mL/min n = 14	Cohort 4^a^ 0.167 mL/min n = 13
AUC_(0–_∞_),_ day×µg/mL	1,426.5	1,243.3	1,251.6	897.0	1,340.1
% CV	30.1	25.8	45.5	39.1	25.7
Fold difference^b^	NA	1.2	1.1	1.6	1.1
95% CI	NA	0.92, 1.43	0.85, 1.53	0.65, 3.87	0.85, 1.33
C_max_, µg/mL	41.7	34.1	36.1	27.0	40.3
% CV	28.2	19.6	35.7	33.9	17.4
t_max_, day, median	8.0	6.0	8.0	6.0	6.0
Minimum, maximum	4, 15	4, 10	4, 10	4, 10	4, 10
AUC_(0–t)_, day×µg/mL	1,154.9	998.1	1014.0	685.7	1,112.4
% CV	25.9	21.9	41.4	35.9	20.7
T_1/2_, day	21.6	22.0	21.9	20.6	20.6
% CV	26.3	24.1	19.3	20.7	21.5
CL/F, mL/day	210.3	241.3	239.7	334.4	223.9
% CV	33.4	29.2	44.3	355.5	25.7
Vz/F, mL	6,560.0	7,658.0	7,577.6	9,944.1	6,666.3
% CV	29.9	30.1	35.2	355.4	19.0

AUC_(0–∞)_ = area under the serum concentration-time curve from zero to infinity; AUC_(0–t)_ = AUC to last observation; CI = confidence interval; CL/F = apparent systemic clearance; C_max_ = maximum concentration; CV = coefficient of variation; NA = not applicable; PK = pharmacokinetic; SC = subcutaneous; t_max_ = time to C_max_; T_1/2_ = half-life; Vz/F = apparent terminal-phase volume of distribution. ^a^Additional analysis excluding the participant with extensive leakage of tralokinumab. ^b^Fold difference is the ratio of the geometric mean AUC_(0–∞)_ relative to cohort 1.

**Table 4.** Summary of reported injection-site reactions within 72 hours of injection of 300 mg tralokinumab (as-treated population).

Injection-site reaction, n	Tralokinumab 300 mg				
	2 × 1 mL SC injections	1 × 2 mL SC injection			
	Cohort 1 6 mL/min n = 15	Cohort 2 12 mL/min n = 15	Cohort 3 2 mL/min n = 15	Cohort 4 0.167 mL/min N = 15	Total n = 60
Participants with ≥ 1 injection-site reaction within 72 h duration^a^	8 (53.3%)	6 (40.0%)	13 (86.7%)	14 (93.3%)	41 (68.3%)
Erythema	7 (46.7%)	5 (33.3%)	12 (80.0%)	11 (73.3%)	35 (58.3%)
Hematoma or bleeding	2 (13.3%)	1 (6.7%)	3 (20.0%)	5 (33.3%)	11 (18.3%)

SC = subcutaneous. ^a^Participants were counted once for any injection-site reaction ≤ 72 h in duration regardless of number of events.

**Table 5.** Overall summary of TEAEs reported (as-treated population).

	Tralokinumab 300 mg				
	2 × 1 mL SC injections	1 × 2 mL SC injection			
	Cohort 1 6 mL/min n = 15	Cohort 2 12 mL/min n = 15	Cohort 3 2 mL/min n = 15	Cohort 4 0.167 mL/min n = 15	Total N = 60
Total number of participants reporting:					
≥ 1 TEAE	5 (33.3%)	5 (33.3%)	4 (26.7%)	4 (26.7%)	18 (30.0%)
≥ 1 treatment-related TEAE	1 (6.7%)	3 (20.0%)	3 (20.0%)	3 (20.0%)	10 (16.7%)
≥ 1 SAE^a^	0	0	0	0	0
≥ 1 TEAE leading to discontinuation of tralokinumab	0	0	0	0	0
Deaths	0	0	0	0	0
TEAEs reported by ≥ 5% participants in any cohort^b^:					
Headache	1 (6.7%)	3 (20.0%)	3 (20.0%)	3 (20.0%)	10 (16.7%)
Nasal congestion	1 (6.7%)	0	1 (6.7%)	1 (6.7%)	3 (5.0%)
Rhinorrhea	2 (13.3%)	0	1 (6.7%)	0	3 (5.0%)
Oropharyngeal pain	0	0	1 (6.7%)	1 (6.7%)	2 (3.3%)
Productive cough	0	1 (6.7%)	0	1 (6.7%)	2 (3.3%)
Sneezing	2 (13.3%)	0	0	0	2 (3.3%)
Throat irritation	0	2 (13.3%)	0	0	2 (3.3%)
Vomiting	0	2 (13.3%)	0	0	2 (3.3%)

SAE = serious adverse event; SC = subcutaneous; TEAE = treatment-emergent adverse event. ^a^SAE criteria: death, life threatening, requiring inpatient hospitalization, prolongation of existing hospitalization, persistent or significant disability/incapacity, important medical event, congenital anomaly/birth defect in the offspring of the participant. ^b^Participants were counted once for each category regardless of the number of events.

## References

[1] May RD Monk PD Cohen ES Manuel D Dempsey F Davis NH Dodd AJ Corkill DJ Woods J Joberty-Candotti C Conroy LA Koentgen F Martin EC Wilson R Brennan N Powell J Anderson IK Preclinical development of CAT-354, an IL-13 neutralizing antibody, for the treatment of severe uncontrolled asthma. Br J Pharmacol. 2012; 166: 177–193. 21895629 10.1111/j.1476-5381.2011.01659.x PMC3415647

[2] Woodruff PG Modrek B Choy DF Jia G Abbas AR Ellwanger A Koth LL Arron JR Fahy JV T-helper type 2-driven inflammation defines major subphenotypes of asthma. Am J Respir Crit Care Med. 2009; 180: 388–395. 19483109 10.1164/rccm.200903-0392OC PMC2742757

[3] Piper E Brightling C Niven R Oh C Faggioni R Poon K She D Kell C May RD Geba GP Molfino NA A phase II placebo-controlled study of tralokinumab in moderate-to-severe asthma. Eur Respir J. 2013; 41: 330–338. 22743678 10.1183/09031936.00223411 PMC3561510

[4] Brightling CE Chanez P Leigh R O’Byrne PM Korn S She D May RD Streicher K Ranade K Piper E Efficacy and safety of tralokinumab in patients with severe uncontrolled asthma: a randomised, double-blind, placebo-controlled, phase 2b trial. Lancet Respir Med. 2015; 3: 692–701. 26231288 10.1016/S2213-2600(15)00197-6

[5] Panettieri RA Brightling C Sjobring U Péterffy A Tornling G Daoud SZ Ranade K Hollis S Colice G STRATOS 1 and 2: considerations in clinical trial design for a fully human monoclonal antibody in severe asthma. Clin Investig (Lond). 2015; 5: 701–711.

[6] Brightling C Wang M Braddock M Nordenmark L Gottlow M Colice G MESOS: considerations in designing a mechanistic study for a biologic used to treat asthma. Clin Investig (Lond). 2015; 5: 713–722.

[7] Busse WW Wang M Gibson J Gottlow M Braddock M Colice G TROPOS: designing a clinical trial to evaluate the oral corticosteroid-sparing effect of a biologic in severe asthma. Clin Investig (Lond). 2015; 5: 723–730.

[8] Dias C Abosaleem B Crispino C Gao B Shaywitz A Tolerability of high-volume subcutaneous injections of a viscous placebo buffer: A randomized, crossover study in healthy subjects. AAPS PharmSciTech. 2015; 16: 1101–1107. 25693652 10.1208/s12249-015-0288-y PMC4674646

[9] Berteau C Filipe-Santos O Wang T Rojas HE Granger C Schwarzenbach F Evaluation of the impact of viscosity, injection volume, and injection flow rate on subcutaneous injection tolerance. Med Devices (Auckl). 2015; 8: 473–484. 26635489 10.2147/MDER.S91019 PMC4646585

[10] Torjman MC Machnicki R Lessin J Loeum C Steinberger D Mycroft S Joseph JI Evaluation of an investigational wearable injector in healthy human volunteers. Expert Opin Drug Deliv. 2017; 14: 7–13. 27809609 10.1080/17425247.2017.1252748

[11] Jørgensen JT Rømsing J Rasmussen M Møller-Sonnergaard J Vang L Musaeus L Pain assessment of subcutaneous injections. Ann Pharmacother. 1996; 30: 729–732. 8826549 10.1177/106002809603000703

[12] Heise T Nosek L Dellweg S Zijlstra E Præstmark KA Kildegaard J Nielsen G Sparre T Impact of injection speed and volume on perceived pain during subcutaneous injections into the abdomen and thigh: a single-centre, randomized controlled trial. Diabetes Obes Metab. 2014; 16: 971–976. 24720741 10.1111/dom.12304

[13] HUMIRA^®^. Adalimumab Prescribing Information. AbbVie Inc., North Chicago, IL 60064, USA. 2015 http://www.rxabbvie.com/pdf/humira.pdf.

[14] STELARA^®^. Ustekinumab Prescribing Information. Janssen Biotech, Inc., Horsham, PA 19044, USA. 2014 http://www.stelarainfo.com/pdf/PrescribingInformation.pdf.

[15] SIMPONI^®^. Golimumab Prescribing Information. Janssen Biotech, Inc., Horsham, PA 19044, USA. 2016 http://www.simponi.com/shared/product/simponi/prescribing-information.pdf.

[16] PRALUENT^®^. Alirocumab Prescribing Information. Sanofi-Aventis US LLC, Bridgewater, NJ 08807, USA. 2015 http://www.regeneron.com/Praluent/Praluent-fpi.pdf.

[17] ENBREL^®^. Etanercept Prescribing Information. Amgen Inc., Immunex Corporation, Thousand Oaks, CA 91320 USA. 20152015 http://pi.amgen.com/united_states/enbrel/derm/enbrel_pi.pdf.

[18] Shire SJ Shahrokh Z Liu J Challenges in the development of high protein concentration formulations. J Pharm Sci. 2004; 93: 1390–1402. 15124199 10.1002/jps.20079

[19] Liu J Nguyen MD Andya JD Shire SJ Reversible self-association increases the viscosity of a concentrated monoclonal antibody in aqueous solution. J Pharm Sci. 2005; 94: 1928–1940. 16052543 10.1002/jps.20347

[20] Jezek J Rides M Derham B Moore J Cerasoli E Simler R Perez-Ramirez B Viscosity of concentrated therapeutic protein compositions. Adv Drug Deliv Rev. 2011; 63: 1107–1117. 22014592 10.1016/j.addr.2011.09.008

[21] Gardulf A Nicolay U Asensio O Bernatowska E Böck A Carvalho BC Granert C Haag S Hernández D Kiessling P Kus J Pons J Niehues T Schmidt S Schulze I Borte M Rapid subcutaneous IgG replacement therapy is effective and safe in children and adults with primary immunodeficiencies – a prospective, multi-national study. J Clin Immunol. 2006; 26: 177–185. 16758340 10.1007/s10875-006-9002-x

[22] Gustafson R Gardulf A Hansen S Leibl H Engl W Lindén M Müller A Hammarström L Rapid subcutaneous immunoglobulin administration every second week results in high and stable serum immunoglobulin G levels in patients with primary antibody deficiencies. Clin Exp Immunol. 2008; 152: 274–279. 18341618 10.1111/j.1365-2249.2008.03620.x PMC2384102

[23] Doughty DV Clawson CZ Lambert W Subramony JA Understanding Subcutaneous Tissue Pressure for Engineering Injection Devices for Large-Volume Protein Delivery. J Pharm Sci. 2016; 105: 2105–2113. 27287520 10.1016/j.xphs.2016.04.009

[24] Wiesenfeld-Hallin Z Sex differences in pain perception. Gend Med. 2005; 2: 137–145. 16290886 10.1016/s1550-8579(05)80042-7

[25] Paller CJ Campbell CM Edwards RR Dobs AS Sex-based differences in pain perception and treatment. Pain Med. 2009; 10: 289–299. 19207233 10.1111/j.1526-4637.2008.00558.x PMC2745644

[26] Oh CK Faggioni R Jin F Roskos LK Wang B Birrell C Wilson R Molfino NA An open-label, single-dose bioavailability study of the pharmacokinetics of CAT-354 after subcutaneous and intravenous administration in healthy males. Br J Clin Pharmacol. 2010; 69: 645–655. 20565456 10.1111/j.1365-2125.2010.03647.x PMC2883757

[27] Jensen MP Chen C Brugger AM Interpretation of visual analog scale ratings and change scores: a reanalysis of two clinical trials of postoperative pain. J Pain. 2003; 4: 407–414. 14622683 10.1016/s1526-5900(03)00716-8

[28] Lobo ED Hansen RJ Balthasar JP Antibody pharmacokinetics and pharmacodynamics. J Pharm Sci. 2004; 93: 2645–2668. 15389672 10.1002/jps.20178

[29] Coghill RC McHaffie JG Yen YF Neural correlates of interindividual differences in the subjective experience of pain. Proc Natl Acad Sci USA. 2003; 100: 8538–8542. 12824463 10.1073/pnas.1430684100 PMC166264

[30] Coghill RC Eisenach J Individual differences in pain sensitivity: implications for treatment decisions. Anesthesiology. 2003; 98: 1312–1314. 12766637 10.1097/00000542-200306000-00003

[31] Nielsen CS Staud R Price DD Individual differences in pain sensitivity: measurement, causation, and consequences. J Pain. 2009; 10: 231–237. 19185545 10.1016/j.jpain.2008.09.010

[32] Baverel PG Jain M Stelmach I She D Agoram B Sandbach S Piper E Kuna P Pharmacokinetics of tralokinumab in adolescents with asthma: implications for future dosing. Br J Clin Pharmacol. 2015; 80: 1337–1349. 26182954 10.1111/bcp.12725 PMC4693499

